# Multifactorial etiology of manic episodes. About a case

**DOI:** 10.1192/j.eurpsy.2024.897

**Published:** 2024-08-27

**Authors:** M. Valverde Barea, F. Vílchez Español, L. Soldado Rodríguez, M. O. Solis Correa

**Affiliations:** ^1^Psychiatry, H.U. Jaén, Jaén; ^2^Psychiatry, USMC Baza, Baza, Spain

## Abstract

**Introduction:**

Manic episodes have a multifactorial etiology, with frequent association with genetic factors, comorbidities such as systemic diseases or secondary to infectious diseases, and environmental exposure factors. The prevalence of bipolar disorder is markedly higher in patients with autoimmune disease. The risk of developing bipolar disorder in some studies has been seen to be higher among patients with rheumatoid arthritis, therefore chronic inflammation would be a potential mechanism and could be a modifiable risk factor for bipolar disorder. Growing evidence indicates that Sars-CoV-2 may also trigger the acute onset of mood disorders or psychotic symptoms.

**Objectives:**

We present the case of a patient who presents symptoms compatible with an acute manic episode after an outbreak of rheumatoid arthritis and comorbid COVID infection.

**Methods:**

52-year-old patient. She went to the hospital emergency room presenting affective symptoms compatible with a manic episode and psychomotor agitation. Personal medical history: rheumatoid arthritis, antiphospholipid syndrome. Psychiatric personal history: Depressive disorder under follow-up by a private psychiatrist under treatment with antidepressants. During the interview, the patient presented accelerated speech, with great emotional incontinence. Saltigrade thought and tachypsychia. She verbalizes delusional ideas of megalomaniacal and mystical and religious characteristics. She verbalizes that she is the reincarnation of the holy spirit, that God has taken her body and speaks through her. In the emergency room, a Sars-CoV-2 infection that the patient was unaware of was diagnosed. She is admitted to the hospital in the mental health unit, in the first interviews the patient maintains speech with delusional ideas “I notice the stigmata of Christ on my body”.

**Results:**

The patient recovers after treatment for the COVID infection, remaining asymptomatic. It was decided to start lithium to stabilize mood and the patient presented good tolerance and treatment with antipsychotics. The patient presented a favorable response, remitting the psychotic symptoms of which she was critical and stabilizing the affective symptoms. The patient is diagnosed with Severe Manic Episode with Psychotic Symptoms, as the main diagnosis and we could conclude the diagnosis of Bipolar Disorder since she has presented 2 depressive episodes in the past that have required treatment and follow-up by psychiatry.

**Conclusions:**

Manic episodes have a multifactorial etiology and require an individualized approach, and comorbid medical conditions must always be assessed in order to establish a therapeutic plan with patients.

**Disclosure of Interest:**

None Declared

